# Strategies to strengthen elective surgery systems during the SARS-CoV-2 pandemic: systematic review and framework development

**DOI:** 10.1093/bjs/znad405

**Published:** 2024-02-01

**Authors:** NIHR Global Health Research Unit on Global Surgery, A O Ademuyiwa, A Bhangu, S Chakrabortee, J Glasbey, S K Kamarajah, V Ledda, E Li, D Morton, D Nepogodiev, M Picciochi, J F F Simoes, M C Lapitan, M Cheetham, E Forkman, E El-Boghdadly, D Ghosh, E M Harrison, P Hutchinson, I Lawani, M L Aguilera, J Martin, J G Meara, F Ntirenganya, A Ramos De-la Medina, S Tabiri

**Affiliations:** NIHR Global Health Research Unit on Global Surgery, Institute of Applied Health Research, University of Birmingham, Birmingham, UK

## Introduction

Surgical systems are complex and susceptible to external system pressures. The SARS-CoV-2 pandemic and its impact on elective surgery illustrated this. The reassignment of resources to the acute care of patients with COVID-19 led to cancellations of elective services that varied according to surges of the pandemic. Overall, it was estimated that more than 28 million operations were cancelled or postponed globally^1^. Waiting lists are now a public health crisis, with anticipated impacts on health outcomes, economies, and societies for an undetermined length of time^2^. For instance, there were approximately 4.3 million patients waiting for surgery in June 2022 in the UK^3^, and 7.5 million waiting for any elective care in England in June 2023. With other factors contributing to more cancellations (for example, climate change, strikes, supply interruptions), the current number is expected to be even higher. Although data are scarce for other countries, similar patterns are expected^4,5^.

A diverse range of adaptations emerged and were scaled from the beginning of the pandemic to support continuation of elective surgery. Although some existed previously, the pandemic provided a unique opportunity to expand approaches to tackle an anticipated growth in all elective surgery waiting lists. To understand the elective surgical system, using the organizational domains described in the National Surgical, Obstetric and Anaesthetic plan (NSOAP) is extremely helpful^6^. NSOAP is a policy process and framework used to comprehensively address the health burden of conditions requiring surgery, and includes infrastructure, workforce, service delivery, financing, information management, and governance as domains^6,7^. Learning adaptations that have been successful will be useful in helping policymakers to decide which strategies would work better in their centre. Adoption of a hub-and-spoke model and performing surgery at weekends are examples of what has been done so far^8,9^. However, a research gap exists on understanding how these strategies can inform planning of elective surgery after the pandemic^2^.

The primary aim of this study was to identify and describe the strategies adopted globally that supported continuation of elective surgery and which can support continuation of elective surgery during times of health system stress. The secondary aim was to characterize the strengths and limitations associated with each strategy.

## Methods

**Fig. 1:**
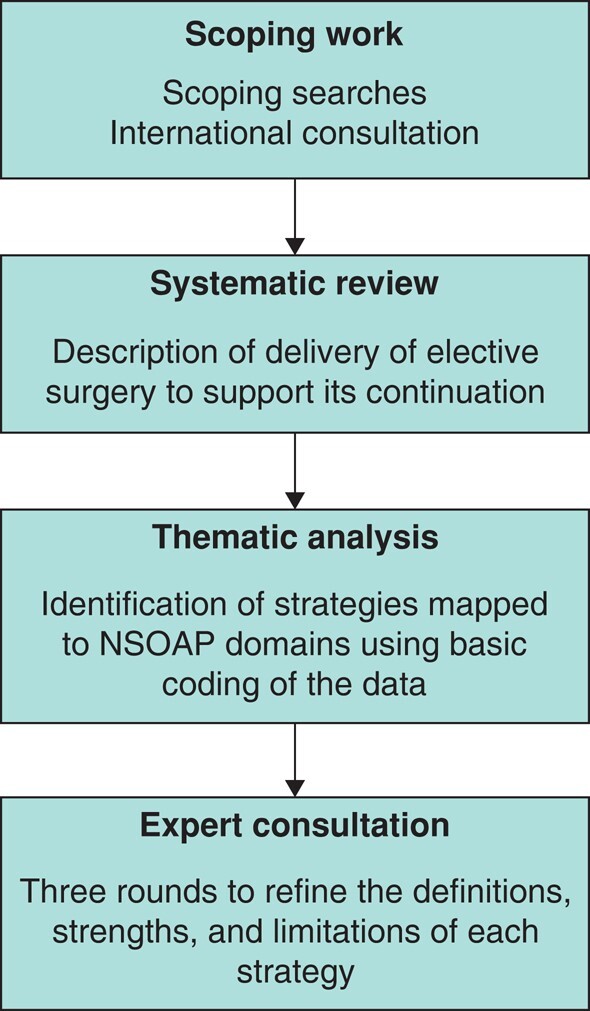
Overview of four phases NSOAP, National Surgical Obstetric and Anaesthesia Planning.

This study included four phases (*Fig. 1*). First, the scoping work included searches and discussion with frontline teams, which informed the depth and concepts of the systematic review. Second, a systematic review identified studies describing adaptations to elective surgery introduced or expanded during the pandemic. Third, thematic content analysis was undertaken to identify mitigation strategies for elective surgery provision. Finally, the strategies were refined through three rounds of expert consensus.

### Phase 1: scoping work

Scoping searches in the published literature and grey literature were undertaken to inform the depth of the systematic review. Grey literature encompasses ‘information produced on all levels of government, academics, business and industry in print and electronic formats, but which is not controlled by commercial publishers’. Searches in PubMed and Google Scholar with search terms related to ‘elective’, ‘surgery’, and ‘adaptations’ were conducted. When any strategies were reported at this stage, they were used as deductive themes during the thematic analysis stage. To develop an overview of the research topic, elective surgery recovery and waiting lists were discussed with a group of frontline surgeons and anaesthetists attending the National Institute for Health Research (NIHR) Global Surgery Unit annual meeting in New Delhi, India, in September 2022. This event was chosen because of the opportunity to discuss in person with the NIHR Global Surgery Unit network. This network is composed of surgeons and anaesthetists from low- and middle-income countries, with selected experts having clinical and managerial leadership roles within their hospitals and/or nationally. All attendees of the meeting were invited by e-mail to participate, and questions were preplanned to guide the discussion (*Table S1*). Sixteen participants were involved in the discussion: one representative each from Nigeria, Benin, South Africa, Rwanda, Ghana, Mexico, and India hubs, eight researchers with previous experience on waiting lists and elective surgery research (5 from the UK, 1 from India, 1 from Guatemala, 1 from the Philippines), and one healthcare consultant from the UK. The meeting was audio recorded and transcribed to identify the key messages from this discussion, which were used to inform the scope of the systematic review.

### Phase 2: systematic review

#### Search strategy

A systematic review was conducted to identify studies reporting adaptations to elective surgical systems during the pandemic that supported the continuation of elective surgery. PRISMA guidelines^10^ were followed and the protocol was registered prospectively (PROSPERO: CRD42023366245). A systematic search was undertaken in Embase and MEDLINE via Ovid on 23 November 2022. Search terms relating to ‘elective’, ‘surgery’, and ‘SARS-CoV-2’ were used. Searches were restricted to between 11 March 2020 and 22 November 2022. The full search strategy adopted can be found in *Table S2*. Grey literature and trial database searches were not conducted.

#### Study selection

The titles and abstracts were screened by two reviewers, and full texts were assessed against the inclusion criteria using Rayyan software^11^. Inconsistencies in the selection process were discussed and resolved with a third reviewer. Elective surgery was defined as any operation that was planned and booked in advance of routine admission to hospital. Strategy was defined as any change or adaptation that supported continuation of elective surgery. Studies that described the delivery of elective surgery during the SARS-CoV-2 pandemic were included. Studies reporting adaptations from any surgical specialty, from any hospital, and from any country were included. Papers that described prioritization tools were excluded as these tools reflect who should have the operation first rather than how to support continuation of elective surgery. Studies that evaluated only operations on patients infected with SARS-CoV-2 were also excluded. Guidelines, recommendations, consensus, and report studies were also excluded because these do not consider the adoption in practice of the strategies suggested.

#### Data extraction and management

From each included study, data extraction included description of the strategy, strengths and limitations acknowledged with that strategy, duration of study, study design, country, specialty, procedures included, and year of the study. The data were extracted and recorded in Microsoft Excel^®^ (Microsoft, Redmond, WA, USA) by the two reviewers, and inconsistencies were discussed and resolved by repeated extraction of the data with further reviews of the full text with a senior researcher. Data describing the adaptation reported in each included paper were kept verbatim to allow further analysis.

### Phase 3: thematic analysis

Thematic content analysis of eligible papers was conducted according to Clarke and Braun’s principles^12^: familiarization; coding; generating initial themes; developing and reviewing themes; refining and naming themes; and writing up. Familiarization was done during the data extraction by highlighting and transcribing verbatim the relevant parts of the paper related to the adaptation described. These were recorded in Microsoft Excel^®^ for Mac^®^ version 16.73. A mixed inductive and deductive thematic analysis with information from the scoping work and the systematic review was conducted by one researcher. A theoretical approach was used, with open coding to code the data, including the segments that were relevant to identify the adaptations to elective surgery. Major themes were identified and refined iteratively to generate a preliminary framework of strategies. These were then discussed and reviewed during weekly meetings within a wider study group. Each strategy was then mapped to one of the NSOAP domains. Proposed strengths and limitations of each strategy were extracted from each paper to inform the expert consultation.

### Phase 4: expert consultation

Expert consensus was gained over three rounds to refine the strategies, inform potential cross-contextual strengths and limitations, and explore where the strategies might improve surgical preparedness, defined using the Surgical Preparedness Index (SPI)^13^. This is a set of 23 core indicators that measure preparedness of elective surgery when pressured by external stressors such as pandemics, seasonal pressures, political conflicts, and natural disasters. Its application allows identification of areas requiring further improvement. Owing to the predefined research aim, three different expert groups were consulted during the consensus process. In round 1, experts were sampled from within the NIHR Global Surgery Unit network, and asynchronous discussions and iterative edits were conducted using a Google Docs^®^ online document (Google, Mountain View, CA, USA). In the second round, the definitions and implementation of included strategies were refined in online meetings of the COVID-19 Recovery Group of the Royal College of Surgeons of England using Zoom^®^ (Zoom Corporation, Tokyo, Japan). This group was composed of surgeons and trainees involved in planning elective surgery and service adaptations in UK hospitals. Finally, experts within the study management group made iterative refinements of the data generated. All three groups reviewed the final framework presented in this study and agreed with the proposed framework.

## Results

### Scoping work

Discussions during the Global Surgery Unit annual meeting contributed to defining the research question and search terms (*Table S3*), by ensuring that the investigated topic was relevant across different contexts. Scoping reviews identified three phases when adaptations were implemented during the SARS-COV-2 pandemic: the preoperative phase, the operative phase, and the postoperative phase. This review and framework development focused on the operative phase, which spanned the interval between time of admission and discharge from hospital after surgery, as it was deemed to be easy to influence with adaptations being rapidly implemented. This phase also had the most variation in adaptation strategies, and warranted exploration and ranking of potentially effective adaptations.

### Study characteristics

**Fig. 2:**
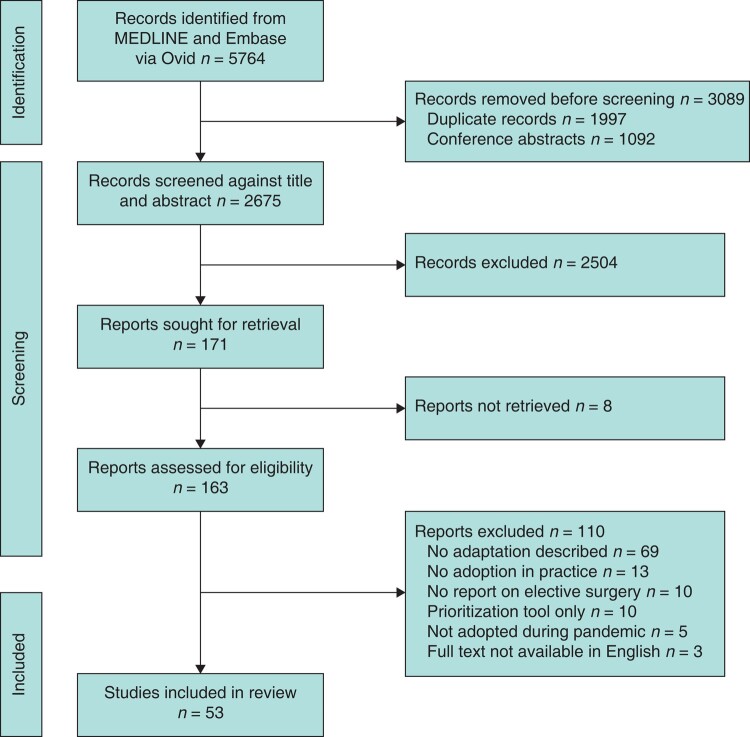
PRISMA flow chart of included studies

A total of 5674 records were identified, from which 163 studies were reviewed, and 53 were included (*Fig. 2*). Most studies were from high-income countries (52 of 53); only 1^14^ was from an upper-middle-income country (China). Most studies reflected the practices adopted in a single country (52 of 53). Countries most frequently represented were the UK (22 of 53), USA (11 of 53), and Italy (9 of 53). Only one study^15^ evaluated data from multiple countries (China, Italy, Spain, and USA) (*Table S4*). There was a lot of variability in the context and specialties of reporting adaptations. Only three studies^16–18^ evaluated an adaptation implemented across all surgical specialties in the participating centres. General surgery (19 of 53) and orthopaedics (19 of 53) were the most frequent specialties reporting adaptations (*Table S5*). The median duration of evaluation of adaptations in included studies was 3.5 months; however, this did not necessarily reflect the duration of the strategy, which may have been longer. There was no cost evaluation reported in any of the included studies. Most studies were interrupted time series (37 of 53), of which 29 had data collected retrospectively and 8 prospectively. There were no randomized trials, and two^9,19^ were quality improvement studies. There were two narrative reviews and one systematic review captured in this project (*Table S4*)^15,20,21^.

### Strategies to strengthen elective surgery

Themes identified in the scoping work were used as deductive themes when coding the data. Three strategies were reported frequently at that stage: standalone hub, integrated hub, and day-case surgical unit. These themes were used as inductive themes^6^, and mapped well to NSOAP domains, so this framework was brought in to guide further analysis.

**Fig. 3:**
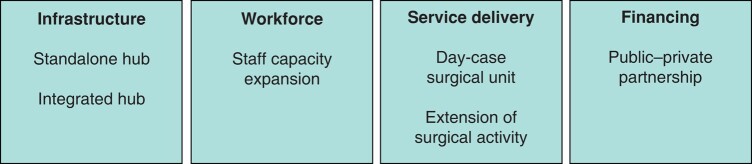
Framework development: strategies mapped to National Surgical Obstetric and Anaesthesia Planning domains

From basic coding of the data, three more themes were identified from the reported adaptations. The six themes were used to define strategies to strengthen elective surgery delivery: standalone hub^15,17,22–38^, integrated hub^39–44^, public–private partnership^14,16,18,20,23,24,44–49^, day-case surgical unit^9,19,21,22,48–63^, extension of surgical activity^9,60^, and staff capacity expansion^18,64^. Each strategy was directly related to four domains from the NSOAP: infrastructure, workforce, service delivery, and financing (*Fig. 3*). Adaptations to infrastructure were reported most frequently (25 of 53), followed by adaptations to service delivery (22 of 53), adaptations to financing (12 of 53), and adaptations to workforce (2 of 53). No papers were identified that reported adaptations to information management and governance.

Strategies were reported to be implemented alone or in combination, as reported in nine studies^9,18,22–24,44,48,49,60^, and a public–private partnership was present in most of them (6 of 9)^18,23,24,44,48,49^. Each strategy was defined based on the characteristics described in the literature (*Table 1*). The most common strategies reported were day-case surgical unit (20 of 53), defined as a surgical unit with a dedicated admission and recovery area, where patients are discharged during the same day; and standalone hub (19 of 53), defined as a dedicated hospital to provide elective care, without acute admissions, that works through a referral pathway among other hospitals.

**Table 1:** Definition of strategies identified

NSOAP domain	Strategies and definition
Infrastructure	Standalone hub Ring-fenced hospital (hospital dedicated to planned surgery only) without acute admissions, which provides operating theatres, wards, +/− critical care beds Able to provide elective surgery to patients from a network of referring hospitals Staff can be recruited from the different referring hospitals with a predefined rota in place It should be able to transfer patients to different hospitals in the event that emergency surgery is needed in postoperative phase If cancer surgery is performed, a different MDT needs to be in place to prioritize patients and a pathology laboratory is also needed as part of hub
Infrastructure	Integrated hub Ring-fenced operating theatre (reserved planned surgery theatres) and ward beds (reserved planned surgery beds) within an acute hospital Ring-fenced staff (reserved teams to provide planned surgical care) that are not allocated to other tasks In the event that patients with cancer have surgery, the hospital should already have its own MDT and pathology laboratory Internal regulation needed to maintain activity throughout the year
Workforce	Staff capacity expansion Transferring surgical skills to junior doctors and/or non-surgeons and/or retired professionals to expand the workforce able to provide a surgical procedure, applied to surgical and anaesthesia team Requires planning types of surgical procedure where this can be adopted as well as a risk prediction score to adapt complexity of patients to level of operator
Service delivery	Extension of surgical activity Ring-fenced operating theatres that are used to perform surgery in extended hours, outside of usual use of theatres, such as evenings and weekends Dedicated staff need to be allocated to these hours of work and contracts might need adjustment To reduce the turnover time between patients, high intensity theatres might be used
Service delivery	Day-case surgical unit Ambulatory surgical unit with allocated operating theatres and dedicated admission and recovery area Staff allocated to this unit need to be free from other tasks
Financing	Public–private partnerships Ring-fenced operating theatre, wards, and ICU beds in a private hospital to provide elective surgery to patients from public sector Staff available from private sector can provide staff capacity expansion Planning includes selection of procedures tailored to capability of staff and facilities in private hospital A contract between public and private hospital to define remit and responsibility is necessary

NSOAP, National Surgical Obstetric and Anaesthesia Planning; MDT, multidisciplinary team.

Evaluating the contexts in which each strategy was adopted, day-case surgical units were mainly reported in orthopaedic surgery, with arthroplasty being the most common procedure performed in this context. Procedure selection, and patient selection and education were recurrent themes in the papers describing day-case surgical units. In contrast, standalone hubs were not associated with a particular surgical specialty, but the procedures undertaken were mainly major surgery or cancer surgery, supporting the ability to perform more complex surgery in this strategy. No specific predominant themes were identified from the other strategies, regarding either surgical specialties or surgical procedure (*Table S5*).

Only 36 of the 53 papers acknowledged the limitations associated with the strategy reported, but all recognized the strengths associated with each strategy (*Table 2*). Strengths and weaknesses of each strategy were also informed by the three rounds of the expert consultation and planning before implementation was considered a key step to support the implementation process. In the first round (*n* = 25), the group comprised decision-makers (8 of 25), and surgeons or anaesthetists who were involved in the management of elective surgery in their hospitals (11 of 25) or were involved in previous research addressing elective surgery and waiting lists (6 of 25). Three-quarters (18 of 25) had previously participated in the discussion during the Global Surgery Unit annual meeting. In the second round, these were discussed in the monthly meetings of the Recovery Group of the Royal College of Surgeons between December 2022 and May 2023, which was composed of 89 surgeons and anaesthetists who were invited to the meetings each month. The strategies were agreed to have different potential impacts on the preparedness of elective surgical services. Thirteen of 23 SPI indicators fitted within the framework boundaries, and the 6 strategies identified can influence these (*Table S6*).

**Table 2:** Limitations and strengths associated with strategy

Strategy	Strengths	Limitations
Standalone hub	Allows highly specialized procedures to be done by the same team, which may improve surgical outcomes Ring-fenced theatres, beds, and staff allow greater resilience to external pressures Improves communication between surgical teams from different hospitals	Referral network of hub and spokes needs to be defined beforehand as well as location of hub considering movement of staff and patients A separate MDT to prioritize patients with cancer from different specialists needs to be arranged if oncology surgery is being performed in hub Complexity of surgical procedures selected to be done in hubs will depend on facilities available for postoperative care (for example inpatient beds, ICU beds) Transfer pathways need to be designed in case emergency reoperation is required If operations are dependent on pathology (such as cancer), transport of samples should be done in the quickest way possible
Integrated hub	Ring-fenced staff makes it more resilient to external pressures Easier emergency care if needed compared with standalone hub	Staff might be reallocated to acute care if there is external pressure (for example, flu season, war) Requires more staff to be employed by hospital to maintain elective activity Complexity of surgical procedures selected to be done in integrated hubs will depend on facilities available for postoperative care (for example inpatient beds, ICU beds)
Staff capacity expansion	Expands training opportunities for surgical trainees	Limited to simple selected procedures that can be performed by junior surgeons A senior surgeon needs to be available to scrub in case support is needed
Extension of surgical activity	Increase in elective lists with more patients being operated per day or week	Available to a limited range of procedures when other services are needed at the same time (for example radiology, pathology) Staff need to be available to work out of hours Reduction in turnover time between patients is supported by limited evidence
Day-case surgical unit	Not dependent on availability of beds Less exposure to hospital-acquired infections for patients operated in this pathway	Requires ending early to allow recovery time to safely discharge patients Planning requires selection of both procedures and patients capable of being discharged on the same day Patient education involves more staff time
Public–private partnership	Allows expansion of infrastructure and staff if private sector provides them	Requires a contract between both sectors to maintain surgical care free from cost to patients The elective surgical care provided is highly dependent on contract conditions between public and private sectors, which may limit care Might depend on staff from public sector to conduct elective procedures in a private setting

ICU, intensive care unit; MDT, multidisciplinary team.

## Discussion

This study identified six strategies to bolster and scale elective surgical capacity that can inform service planning. These proved feasible during the pandemic, and can be used by frontline teams to improve resilience and adaptability in the planned care pathways for surgery in the postpandemic era. The dissemination of these strategies is needed considering the efforts towards increasing elective surgical capacity and efficiency. Mapping these to the NSOAP domains will allow a more focused approach to promoting change. Planning around these strategies will support an increase in elective capacity, but also resilience against future pandemics and other external threats (for example climate change).

One key message from this review is that there is no single solution to all surgical specialties providing elective care. This is supported by the small number of studies that mentioned one strategy adopted across all the different surgical specialties available in that hospital. Implementing each strategy has its challenges, although standalone hubs might work better when there is already a network of hospitals and one of them is designated to provide only elective care. Day-case surgery can be implemented in virtually any hospital, but relies on successful patient selection and education^50^. When planning adoption of the different strategies, it is important to recognize that workforce and financing domains underpin the ability of hospitals to implement change. Although these were under-reported in the literature reviewed, they should be brought to planning discussions.

Research on strengthening and optimizing elective surgery is still in its early stages. Evaluation of the safety and cost-efficiency associated with each strategy reported is still required. An evaluation of the resources that are needed, and how to overcome resource restrictions, will also be relevant and was outside of the scope of this study. A quality improvement project and process evaluation might be two study types to consider. The authors have suggested how these strategies might increase the resilience of the surgical systems using the SPI score, which points to an area for future research^13^.

Limitations associated with this study need to be acknowledged. The short duration of the included studies and the fact that most were cohort studies limit not only the evaluation of the efficacy of the strategies on a long-term basis but also the ability to fully recognize limitations of the strategies in each paper. The lack of a cost evaluation in publications to date is problematic because cost is an essential consideration for policymakers and governments when implementing service change, particularly considering the high cost of surgery. There is a risk of publication bias in the published literature, as changes to elective surgery delivery that were not successful may not have been reported. Furthermore, although broad search terms were included, it is possible that some papers in which relevant details were reported were not identified when the abstract was reviewed at the screening stage. The lack of studies from low- and middle-income countries is also a limitation, which challenges the generalization of these findings to the global context. However, to overcome this, representatives from low- and middle-income countries were included during the expert consultation rounds, when refinement of definitions, strengths, and limitations was undertaken. Finally, the groups from which expert consultation was sought were self-selected, and there were no managers and nurses involved; the latter could add other different perspectives to the topic.

## Collaborators

Writing group: A. O. Ademuyiwa (University of Lagos, Lagos, Nigeria), A. Bhangu, S. Chakrabortee, J. Glasbey, S. K. Kamarajah, V. Ledda, E. Li, D. Morton, D. Nepogodiev, M. Picciochi (first author), J. F. F. Simoes (University of Birmingham, Birmingham, UK), M. C. Lapitan (University of the Philippines Manila, Manila, Philippines), M. Cheetham (Shrewsbury and Telford Hospital NHS Trust, Shrewsbury, UK), E. Forkman (Lund University, Lund, Sweden), K. El-Boghdadly (King's College London, London, UK), D. Ghosh (Christian Medical College, Ludhiana, India), E. M. Harrison (University of Edinburgh, Edinburgh, UK), P. Hutchinson (University of Cambridge, Cambridge, UK), I. Lawani (University of Abomey Calavi, Cotonou, Benin), M. L. Aguilera (Universidad Francisco Marroquín, Guatemala City, Guatemala), J. Martin (University of Western Ontario, Ontario, Canada), J. G. Meara (Harvard Medical School, Boston, USA), F. Ntirenganya (University of Rwanda, Kigali, Rwanda), A. Ramos De-la Medina (Hospital Español de Veracruz, Veracruz, Mexico), S. Tabiri (Tamale Teaching Hospital, Tamale, Ghana) (listed alphabetical by last name).

## Supplementary Material

znad405_Supplementary_Data

## Data Availability

The data presented in this study can be requested by e-mail to the corresponding author.

## References

[1] COVIDSurg Collaborative . Elective surgery cancellations due to the COVID-19 pandemic: global predictive modelling to inform surgical recovery plans. Br J Surg 2020;107:1440–1449 32395848 10.1002/bjs.11746 PMC7272903

[2] Blythe N Ross S . Strategies to reduce waiting times for elective care. *The King's Fund.* www.kingsfund.org.uk/publications/strategies-reduce-waiting-times-elective-care (accessed 15 December 2022)

[3] Nepogodiev D , Acharya R, Chaudhry D et al Forecasting waiting lists for elective procedures and surgery in England: a modelling study. medRxiv 2022. DOI: 10.1101/2022.06.20.22276651

[4] Latijnhouwers D , Pedersen A, Kristiansen E, Cannegieter S, Schreurs BW, van den Hout W et al No time to waste; the impact of the COVID-19 pandemic on hip, knee, and shoulder arthroplasty surgeries in the Netherlands and Denmark. Bone Jt Open 2022;3:977–990 36537253 10.1302/2633-1462.312.BJO-2022-0111.R1 PMC9783280

[5] Van Wyngaard T , Cairncross L, Maswime S, Roodt L, Malherbe F. Impact of COVID-19 on breast cancer diagnostic and surgical services at a South African academic hospital. S Afr J Surg 2022;60:119–123 35851366

[6] Truche P , Shoman H, Reddy CL, Jumbam DT, Ashby J, Mazhiqi A et al Globalization of national surgical, obstetric and anesthesia plans: the critical link between health policy and action in global surgery. Global Health 2020;16:1 31898532 10.1186/s12992-019-0531-5 PMC6941290

[7] Meara JG , Leather AJ, Hagander L, Alkire BC, Alonso N, Ameh EA et al Global surgery 2030: evidence and solutions for achieving health, welfare, and economic development. Lancet 2015;386:569–624 25924834 10.1016/S0140-6736(15)60160-X

[8] McNally S . Scarlett McNally: tackling a huge surgical waiting list needs a different approach. BMJ 2023;380:162 36696971 10.1136/bmj.p162

[9] Matava C , So J, Williams RJ, Kelley S, Group OR-X. A Canadian weekend elective pediatric surgery program to reduce the COVID-19-related backlog: operating room ramp-up after COVID-19 lockdown ends—extra lists (ORRACLE-Xtra) implementation study. JMIR Perioper Med 2022;5:e35584 34887242 10.2196/35584 PMC8929408

[10] Page MJ , McKenzie JE, Bossuyt PM, Boutron I, Hoffmann TC, Mulrow CD et al The PRISMA 2020 statement: an updated guideline for reporting systematic reviews. BMJ 2021;372:n71 33782057 10.1136/bmj.n71 PMC8005924

[11] Ouzzani M , Hammady H, Fedorowicz Z, Elmagarmid A. Rayyan—a web and mobile app for systematic reviews. Syst Rev 2016;5:210 27919275 10.1186/s13643-016-0384-4 PMC5139140

[12] Clarke V , Braun V. Successful Qualitative Research: A Practical Guide for Beginners. UK: Sage, 2013

[13] NIHR Global Health Unit on Global Surgery, COVIDSurg Collaborative . Elective surgery system strengthening: development, measurement, and validation of the surgical preparedness index across 1632 hospitals in 119 countries. Lancet 2022;400:1607–1617 36328042 10.1016/S0140-6736(22)01846-3 PMC9621702

[14] Ho MK . Total joint replacement surgeries: making the case for a public–private partnership in Hong Kong. World Med Health Policy 2021;14:600–608

[15] Hanrahan JG , Burford C, Adegboyega G, Nicolaides M, Boyce L, Wong K et al Early responses of neurosurgical practice to the coronavirus disease 2019 (COVID-19) pandemic: a rapid review. World Neurosurg 2020;141:e1017–e1026 32599184 10.1016/j.wneu.2020.06.167 PMC7318941

[16] Friebel R , Fistein J, Maynou L, Anderson M. Emergency contracting and the delivery of elective care services across the English National Health Service and independent sector during COVID-19: a descriptive analysis. BMJ Open 2022;12:e055875 10.1136/bmjopen-2021-055875 PMC9296998 35851029

[17] AlShareef Y , AlShammary SA, Abuzied Y, AlAsseri Y, AlQumaizi KI. Assigning green hospitals during the COVID-19 pandemic assure continuous and safe resumption of surgical services. Ann Med Surg (Lond) 2022;73:103207 34956643 10.1016/j.amsu.2021.103207 PMC8690220

[18] McCabe R , Schmit N, Christen P, D’Aeth JC, Løchen A, Rizmie D et al Adapting hospital capacity to meet changing demands during the COVID-19 pandemic. BMC Med 2020;18:329 33066777 10.1186/s12916-020-01781-w PMC7565725

[19] Peacock S , Wolfstadt J, Peer M, Gleicher Y. Rapid implementation of an outpatient arthroplasty care pathway: a COVID-19-driven quality improvement initiative. BMJ Open Qual 2022;11:e001698 10.1136/bmjoq-2021-001698 PMC8943481 35318244

[20] Sairally BZF , Clark TJ. Prioritisation of outpatient appointments and elective surgery in gynaecology. Best Pract Res Clin Obstet Gynaecol 2021;73:2–11 33883091 10.1016/j.bpobgyn.2021.03.002 PMC7970415

[21] Thompson JW , Wignadasan W, Ibrahim M, Plastow R, Beasley L, Haddad FS. The introduction of day-case total knee arthroplasty in a national healthcare system: a review of the literature and development of a hospital pathway. Surgeon 2022;20:103–114 33766461 10.1016/j.surge.2021.01.017

[22] Yeung T , Merchant J, Chen P, Smart C, Ghafoor H, Woodhouse F et al The impact and restoration of colorectal services during the coronavirus disease 2019 pandemic: a view from Oxford. Surg Pract 2022;26:27–33 34899957 10.1111/1744-1633.12531 PMC8652538

[23] Chong S , Hung R, Gwozdz A, Irwin S, Eastbury J, Cross T et al 30-Day postoperative COVID-19 outcomes in 398 patients from regional hospitals utilising a designated COVID-19 minimal surgical site pathway. Ann R Coll Surg Engl 2021;103:395–403 33956529 10.1308/rcsann.2020.7072 PMC10335038

[24] Perrone AM , Dondi G, Giunchi S, De Crescenzo E, Boussedra S, Tesei M et al COVID-19 free oncologic surgical hub: the experience of reallocation of a gynecologic oncology unit during pandemic outbreak. Gynecol Oncol 2021;161:89–96 33223219 10.1016/j.ygyno.2020.09.030 PMC7832928

[25] Chu F , Zocchi J, De Berardinis R, Bandi F, Pietrobon G, Scaglione D et al COVID-19 and head and neck cancer management. Experience of an oncological hub comprehensive cancer centre and literature review. Acta Otorhinolaryngol Ital 2022;42:S79–S86 35763278 10.14639/0392-100X-suppl.1-42-2022-09 PMC9137385

[26] Phelan L , Digne-Malcolm H, Hassett D, Naumann DN, Dilworth MP, Bowley DM. Establishing a COVID-secure site for elective surgery during the COVID pandemic: an observational study. J Perioper Pract 2022;33:171–175 35322710 10.1177/17504589211031083 PMC10247672

[27] Milito P , Asti E, Resta M, Bonavina L. Minimally invasive esophagectomy for cancer in COVID hospitals and oncological hubs: are the outcomes different? Eur Surg 2022;54:98–103 35317311 10.1007/s10353-022-00751-1 PMC8932092

[28] Carvalho F , Rogers AC, Chang TP, Chee Y, Subramaniam D, Pellino G et al Feasibility and usability of a regional hub model for colorectal cancer services during the COVID-19 pandemic. Updates Surg 2022;74:619–628 35239150 10.1007/s13304-022-01264-y PMC8891734

[29] Chuntamongkol R , Meen R, Nash S, Ohly NE, Clarke J, Holloway N. Resuming elective orthopaedic services during the COVID-19 pandemic: our experience. Bone Jt Open 2021;2:951–957 34783253 10.1302/2633-1462.211.BJO-2021-0080.R1 PMC8636296

[30] Craig JE , Martin-Krajewski CA, Bledsoe JM, Wensink LJ, Crawford NS, Eberhardt AM et al Regional specialty surgical practice efficiencies gained as a result of COVID-19. Mayo Clin Proc Innov Qual Outcomes 2021;5:693–699 34151194 10.1016/j.mayocpiqo.2021.06.003 PMC8205250

[31] Kasivisvanathan R , Tilney HS, Jhanji S, O’Mahony M, Gruber P, Nicol D et al The ‘hub and spoke model’ for the management of surgical patients during the COVID-19 pandemic. Int J Health Plann Manage 2021;36:1397–1406 34046937 10.1002/hpm.3243 PMC8239827

[32] Spinelli A , Carvello M, Carrano FM, Pasini F, Foppa C, Taffurelli G et al Reduced duration of stay after elective colorectal surgery during the peak phase of COVID-19 pandemic: a positive effect of infection risk awareness? Surgery 2021;170:558–562 33714617 10.1016/j.surg.2020.12.017 PMC7757347

[33] Lee G , Clough OT, Walker JA, Anakwe RE. The perception of patient safety in an alternate site of care for elective surgery during the first wave of the novel coronavirus pandemic in the United Kingdom: a survey of 158 patients. Patient Saf Surg 2021;15:11 33712059 10.1186/s13037-021-00284-8 PMC7952499

[34] Dafydd D A , O’Mahony M, Jhanji S, Devaraj A, Allum W, Nicol D et al The role of CT chest in screening for asymptomatic COVID-19 infection in self-isolating patients prior to elective oncological surgery: findings from a UK Cancer Hub. Br J Radiol 2021;94:20200994 33242245 10.1259/bjr.20200994 PMC7774707

[35] Chessa M , Varrica A, Andronache A, Carminati M, Colli AM, D’Aiello AF et al Lombardy regional urgent reorganization for congenital cardiac patients following the Covid-19 pandemic. J Cardiovasc Med (Hagerstown) 2020;21:654–659 32740498 10.2459/JCM.0000000000001055

[36] Sorrentino L , Guaglio M, Cosimelli M. Elective colorectal cancer surgery at the oncologic hub of Lombardy inside a pandemic COVID-19 area. J Surg Oncol 2020;122:117–119 32476133 10.1002/jso.26052 PMC7300572

[37] Maria G A , Vasileios P, Giacomo Piero I, Ai Ling Loredana R, Marco R, Giulia G et al Urologic surgery and invasive procedures during coronavirus pandemic: retrospective comparison of risk infection in a referral Covid hospital and in a free-Covid hospital. Urologia 2020;87:391560320927106 10.1177/0391560320927106 PMC7549281 32390523

[38] Doyle JP , Patel PH, Doran SLF, Jiao LR, Cunningham D, Nicol D et al The cancer hub approach for upper gastrointestinal surgery during COVID-19 pandemic: outcomes from a UK cancer centre. Ann Surg Oncol 2023;30:2266–2275 36258058 10.1245/s10434-022-12571-4 PMC9579643

[39] Veraldi GF , Mezzetto L, Perilli V, Mastrorilli D, Moratello I, Macrì M et al Clinical and economic impact of COVID-19 in vascular surgery at a tertiary university ‘hub’ hospital of Italy. Ann Vasc Surg 2022;83:97–107 35247541 10.1016/j.avsg.2022.02.004 PMC8889731

[40] Mamidanna R , Askari A, Patel K, Adil MT, Jain V, Jambulingam P et al Safety and feasibility of resuming bariatric surgery under the cloud of COVID-19. Ann R Coll Surg Engl 2021;103:524–529 34192498 10.1308/rcsann.2021.0053 PMC10751989

[41] Huddy JR , Crockett M, Nizar AS, Smith R, Malki M, Barber N et al Experiences of a ‘COVID protected’ robotic surgical centre for colorectal and urological cancer in the COVID-19 pandemic. J Robot Surg 2022;16:59–64 33570736 10.1007/s11701-021-01199-3 PMC7877309

[42] Huddy JR , Freeman Z, Crockett M, Hadjievangelou N, Barber N, Gerrard D et al Establishing a ‘cold’ elective unit for robotic colorectal and urological cancer surgery and regional vascular surgery following the initial COVID-19 surge. Br J Surg 2020;107:e466–e467 32790172 10.1002/bjs.11922 PMC7436658

[43] Pelle F , Cappelli S, Graziano F, Piarulli L, Cavicchi F, Magagnano D et al Breast cancer surgery during the Covid-19 pandemic: a monocentre experience from the Regina Elena National Cancer Institute of Rome. J Exp Clin Cancer Res 2020;39:171 32854728 10.1186/s13046-020-01683-y PMC7450921

[44] Jeannon JP , Simo R, Oakley R, Townley W, Orfaniotis G, Fry A et al Head and neck cancer surgery during the coronavirus pandemic: a single-institution experience. J Laryngol Otol 2021;135:168–172 33517925 10.1017/S0022215121000426 PMC7870905

[45] Prokopenko M , Khatkar H. A district general hospital trauma service response to COVID-19: lessons learnt. Cureus 2020;12:e12087 33489505 10.7759/cureus.12087 PMC7805533

[46] Barker T , Barker P, Sokalsky L, McNamara I. The use of the independent sector in providing NHS services during the Covid-19 outbreak; two hospitals experience. Surgeon 2021;19:e213–e216 33172728 10.1016/j.surge.2020.09.009 PMC7552973

[47] Collins PM , Madden A, O’Connell C, Omer SA, Shakeel Inder M, Casey RG et al Urological service provision during the COVID-19 period: the experience from an Irish tertiary centre. Ir J Med Sci 2021;190:455–460 32856269 10.1007/s11845-020-02352-x PMC7451224

[48] Iqbal MR , Dhahri AA, Darwish NMM, Vijay V. Single centre concept of ‘cold site’ elective surgery during the peak of COVID-19 pandemic: a cohort study. Ann Med Surg (Lond) 2020;59:245–250 33042534 10.1016/j.amsu.2020.09.047 PMC7537605

[49] Hampton M , Riley E, Garneti N, Anderson A, Wembridge K. The orthopaedic waiting list crisis: two sides of the story. Bone Jt Open 2021;2:530–534 34261360 10.1302/2633-1462.27.BJO-2021-0044.R1 PMC8325974

[50] Hammerberg EM , Tucker NJ, Stacey SC, Mauffrey C, Heare A, Verduzco LA et al Institution of same-day total joint replacement at an urban safety net hospital during the COVID-19 pandemic. J Orthop 2022;34:173–177 36060728 10.1016/j.jor.2022.08.029 PMC9422337

[51] Tran-McCaslin M , Basam M, Rudikoff A, Thuraisingham D, McLemore EC. Reduced opioid use and prescribing in a same day discharge pilot enhanced recovery program for elective minimally invasive colorectal surgical procedures during the COVID-19 pandemic. Am Surg 2022;88:2572–2578 35771192 10.1177/00031348221109467 PMC9253719

[52] Cannon DJ , Lewis S, Garcia J, Watkins A, Rodriguez HC, Levy JC. A comparison of patient same-day discharge selection after shoulder arthroplasty before and after the COVID-19 pandemic. Semin Arthroplasty 2022;32:559–563 35431519 10.1053/j.sart.2022.02.011 PMC8994252

[53] Gordon AM , Sheth B, Conway C, Magruder M, Sadeghpour R, Choueka J. The resiliency of elective total shoulder arthroplasty case volumes in the United States during the COVID-19 pandemic: a nationwide temporal trends analysis. J Shoulder Elbow Surg 2022;31:e507–e517 35430366 10.1016/j.jse.2022.02.045 PMC9007746

[54] Seetharam A , Ghosh P, Prado R, Badman BL. Trends in outpatient shoulder arthroplasty during the COVID-19 (coronavirus disease 2019) era: increased proportion of outpatient cases with decrease in 90-day readmissions. J Shoulder Elbow Surg 2022;31:1409–1415 35091073 10.1016/j.jse.2021.12.031 PMC8789381

[55] Cherry A , Montgomery S, Brillantes J, Osborne T, Khoshbin A, Daniels T et al Converting hip and knee arthroplasty cases to same-day surgery due to COVID-19. Bone Jt Open 2021;2:545–551 34293911 10.1302/2633-1462.27.BJO-2021-0029.R1 PMC8325973

[56] Vogel T , Schippers D, Aldarweesh B, Pergolini I, Stollreiter M, Wagner K et al Effective operating room (OR) utilization by performing low-complex surgical procedures during the 2020 corona pandemic. Int J Comput Assist Radiol Surg 2021;16:1357–1359 33999336 10.1007/s11548-021-02392-3 PMC8126596

[57] Brown M , Eardley S, Ahmad J, Lista F, Barr S, Mulholland S et al The safe resumption of elective plastic surgery in accredited ambulatory surgery facilities during the COVID-19 pandemic. Aesthet Surg J 2021;41:NP1427–NP1433 33367485 10.1093/asj/sjaa311 PMC7799347

[58] Boettner F , Bostrom MP, Figgie M, Gonzalez Della Valle A, Haas S, Mayman D et al Timeline and procedures on restarting non-emergent arthroplasty care in the US epicenter of the COVID-19 pandemic. HSS J 2020;16:146–152 33013245 10.1007/s11420-020-09801-4 PMC7524030

[59] Saravana-Bawan B , Auguste BL, Zahirieh AA, Devon K. Ambulatory Parathyroidectomy for Secondary Hyperparathyroidism at a Large Dialysis Program in Toronto: A Program Report. Can J Kidney Health Dis. 2022. DOI: 10.1177/20543581221127937 PMC9619272 36325262

[60] Beninato T , Laird AM, Graves CE, Drake FT, Alhefdhi A, Lee JA et al Impact of the COVID-19 pandemic on the practice of endocrine surgery. Am J Surg 2022;223:670–675 34315576 10.1016/j.amjsurg.2021.07.009 PMC8294714

[61] Grubbs JE , Daigle HJ, Shepherd M, Heidel RE, Kleppe KL, Mancini ML et al Fighting the obesity pandemic during the COVID-19 pandemic. Surg Endosc 2023;37:4895–4901 36163563 10.1007/s00464-022-09628-6 PMC9512967

[62] Magruder ML , Gordon AM, Sheth BK, Conway CA, Wong CHJ. The effects of the COVID-19 pandemic on elective unicompartmental knee arthroplasty in the USA: further evidence that outpatient arthroplasty is safe and effective. Eur J Orthop Surg Traumatol 2023;33:2027–2034 36114876 10.1007/s00590-022-03393-x PMC9483408

[63] Abdelaal MS , Small I, Sherman MB, Courtney PM, Sharkey PF. One year later: the lasting effect of the COVID-19 pandemic on elective hip and knee arthroplasty. J Am Acad Orthop Surg 2022;30:e1474–e1482 36084330 10.5435/JAAOS-D-22-00245

[64] Lin PF , Naveed H, Eleftheriadou M, Purbrick R, Zarei Ghanavati M, Liu C. Cataract service redesign in the post-COVID-19 era. Br J Ophthalmol 2021;105:745–750 32703783 10.1136/bjophthalmol-2020-316917

