# Supplementary material for: Strategies to strengthen elective surgery systems during the SARS-CoV-2 pandemic: systematic review and framework development
Source: Br J Surg. 2024 Feb 1;111(2):znad405. doi: 10.1093/bjs/znad405 (PMC10833142; doi:10.1093/bjs/znad405)
Supplement: znad405_Supplementary_Data [file znad405_supplementary_data.docx]

**Strategies to strengthen elective surgery systems during the SARS-CoV-2 pandemic: a systematic review and framework development**

*NIHR Global Health Research Unit on Global Surgery*

**Corresponding Author**

Dr Maria Picciochi, MD, Clinical Research Fellow in Global Surgery,

NIHR Global Health Research Unit on Global Surgery, University of Birmingham, Institute of Applied Health Research, Heritage Building, Mindelsohn Way, Birmingham, United Kingdom, B15 2TH. Email: [m.picciochi@bham.ac.uk](file:///C:\Users\Maria\Downloads\m.picciochi@bham.ac.uk)

**Supplementary Materials – Index**

| **Supplementary tables** | **Pages** |
| --- | --- |
| Table S1: Pre-planned questions and topics to guide GSU annual meeting discussion | 1 |
| Table S2: Search strategy | 2 |
| Table S3: Key messages from GSU annual meeting | 3 |
| Table S4: Description of included studies | 4-5 |
| Table S5: Strategies, specialties and procedures performed | 6-9 |
| Table S6: Strengthening of system preparedness indicators by this framework | 10 |
| Full list of references | 11-14 |

Table S1: Pre-planned questions and topics to guide GSU annual meeting discussion

| **Waiting lists:**   - How do we define a waiting list for elective surgery? - Are waiting lists for elective surgery a problem in your hospital? |
| --- |
| **Elective surgery adaptations:**   - What has changed in elective surgery in your hospital during the pandemic? - Would be useful to have information about exploring adaptations in physical areas, scheduling, and staff of elective surgery for your contexts? - How can we take the problem of elective surgery to policy makers? |

GSU – NIHR Global Health Research Unit on Global Surgery

Table S2: Search strategy

| **Nr** | **Search terms** | **Results** |
| --- | --- | --- |
| 1 | surg*.ti,ab | 5145470 |
| 2 | Limit 1 to yr= ”2020-Current” | 830428 |
| 3 | operation.ti.ab | 870856 |
| 4 | Limit 3 to yr= ”2020-Current” | 115451 |
| 5 | elective.ti.ab | 228307 |
| 6 | Limit 5 to yr= ”2020-Current” | 41732 |
| 7 | planned.ti.ab | 266137 |
| 8 | Limit 7 to yr= ”2020-Current” | 54833 |
| 9 | programmed.ti.ab | 153648 |
| 10 | Limit 9 to yr= ”2020-Current” | 35969 |
| 11 | scheduled.ti.ab | 159247 |
| 12 | Limit 11 to yr= ”2020-Current” | 29675 |
| 13 | coronavirus.ti.ab | 206459 |
| 14 | Limit 13 to yr= ”2020-Current” | 186767 |
| 15 | covid-19.ti.ab | 524881 |
| 16 | Limit 14 to yr= ”2020-Current” | 524849 |
| 17 | SARS-CoV-2.ti.ab | 181260 |
| 18 | Limit 17 to yr= ”2020-Current” | 181234 |
| 19 | 2 or 4 | 896520 |
| 20 | 6 or 8 or 10 or 12 | 156234 |
| 21 | 14 or 16 or 18 | 597185 |
| 22 | 19 and 20 and 21 | 5764 |

Table S3: Key messages from GSU annual meeting

| **Waiting lists:**   - Waiting lists for elective surgery can be defined as the patients waiting for a planned surgery that had already a decision to surgery. However, there is a ‘hidden’ group of patients that need a planned surgery but might not have a diagnosis yet and/or were not able to access surgical care. - Waiting lists are an unrecognised problem in Ghana, Rwanda, India, Mexico, and South Africa considering the main problem remains in the ‘hidden’ waiting list. This includes all patients that need an operation but are not able to be assessed by a surgeon and/or not have a diagnosis of a surgical disease yet. This reflects the challenges and limitations in accessing surgical care and constitute an unmeasured problem. - Waiting lists defined as the patients waiting for surgery after having a decision for surgery are not perceived as a problem across all settings in Ghana, with a difference between hospitals located in cities and in rural areas. |
| --- |
| **Elective surgery adaptations and discussion:**   - Availability of staff is perceived as the main constraint in limiting elective operations associated which is associated with a low number of workforce available in Ghana and South Africa. - Conducting extra lists during the weekend and establishing public-private partnerships were two strategies adopted in South Africa to maintain elective surgery during the pandemic. - A study conducted in India showed that bed capacity was one the main factor that limits elective surgery. - Identifying adaptations and learning from what has been working in other hospitals was perceived as a relevant research topic. |

GSU – NIHR Global Health Research Unit on Global Surgery

Table S4: Description of included studies

| **Author of the study** | **Study design** | **Country** | **Year** |
| --- | --- | --- | --- |
| *Lee et al ^1^* | cross sectional study | UK | 2020 |
| *Ong et al^2^* | cross-sectional study | Australia | 2020 |
| *Beninato et al^3^* | cross-sectional study | USA | 2020 |
| *Chessa et al^4^* | cross-sectional study | Italy | 2020 |
| *Boettner et al^5^* | letter | USA | 2020 |
| *Huddy et al (a)^6^* | letter | UK | 2020 |
| *Lin et al^7^* | letter | UK | 2020 |
| *Sorrentino et al^8^* | letter | Italy | 2020 |
| *Saravana-Bawan et al^9^* | letter | Canada | 2021 |
| *Margaret K. Ho^10^* | letter | China | 2020 |
| *Kasivisvanathan et al^11^* | letter | UK | 2020 |
| *Chuntamongkol et al^12^* | prospective observational study | UK | 2020 |
| *Mamidanna et al^13^* | prospective observational study | UK | 2020 |
| *Spinelli et al^14^* | prospective observational study | Italy | 2020 |
| *Brown et al^15^* | prospective observational study | Canada | 2020 |
| *Perrone et al^16^* | prospective observational study | Italy | 2020 |
| *Collins et al^17^* | prospective observational study | Ireland | 2020 |
| *Doyle et al^18^* | prospective observational study | UK | 2020 |
| *Grubbs et al^19^* | prospective observational study | USA | 2020 |
| *Peacock et al^20^* | quality improvement study | Canada | 2021 |
| *Matava et al^21^* | quality improvement study | Canada | 2021 |
| *Hammerberg et al^22^* | retrospective and prospective observational study | USA | 2020 |
| *Friebel et al^23^* | retrospective observational study | UK | 2020 |
| *Tran-McCaslin et al^24^* | retrospective observational study | USA | 2020 |
| *Chu et al^25^* | retrospective observational study | Italy | 2020 |
| *Cannon et al^26^* | retrospective observational study | USA | 2020 |
| *Gordon et al^27^* | retrospective observational study | USA | 2020 |
| *Phelan et al^28^* | retrospective observational study | UK | 2020 |
| *Milito et al^29^* | retrospective observational study | Italy | 2020 |
| *Veraldi et al^30^* | retrospective observational study | Italy | 2020 |
| *Carvalho et al^31^* | retrospective observational study | UK | 2020 |
| *Seetharam et al^32^* | retrospective observational study | USA | 2020 |
| *AlShareef et al^33^* | retrospective observational study | Saudi Arabia | 2020 |
| *Yeung et a^34^l* | retrospective observational study | UK | 2020 |
| *Cherry et al^35^* | retrospective observational study | Canada | 2020 |
| *Craig et al^36^* | retrospective observational study | USA | 2020 |
| *Vogel et al^37^* | retrospective observational study | Germany | 2020 |
| *Chong et al^38^* | retrospective observational study | UK | 2020 |
| *Huddy et al (b)^39^* | retrospective observational study | UK | 2020 |
| *Jeannon et al^40^* | retrospective observational study | UK | 2020 |
| *Prokopenko and Khatkar^41^* | retrospective observational study | UK | 2020 |
| *Dafydd et al^42^* | retrospective observational study | UK | 2020 |
| *Barker et al^43^* | retrospective observational study | UK | 2020 |
| *McCabe et al^44^* | retrospective observational study | UK | 2020 |
| *Rafaih Iqbal et al^45^* | retrospective observational study | UK | 2020 |
| *Pelle et al^46^* | retrospective observational study | Italy | 2020 |
| *Antonio Maria et al^47^* | retrospective observational study | Italy | 2020 |
| *Hampton et al^48^* | retrospective observational study | UK | 2020 |
| *Magruder et al^49^* | retrospective observational study | USA | 2020 |
| *Abdelaal et al^50^* | retrospective observational study | USA | 2020 |
| *Thompson et al^51^* | review | UK | 2020 |
| *Sairally and Clark^52^* | review | UK | 2020 |
| *Hanrahan et al^53^* | systematic review | multiple | 2020 |

Table S5 – Strategies, specialties, and procedures performed

| **Author of the study** | **Strategies** | **Specialties** | **Procedures performed** |
| --- | --- | --- | --- |
| *Hammerberg et al^22^* | Day case surgical unit | Orthopaedic surgery | Knee and hip replacement |
| *Tran-McCaslin et al^24^* | Day case surgical unit | General surgery | Colorectal surgery (MIS) |
| *Cannon et al^26^* | Day case surgical unit | Orthopaedic surgery | Total shoulder arthroplasty or reverse shoulder arthroplasty |
| *Gordon et al^27^* | Day case surgical unit | Orthopaedic surgery | Total shoulder arthroplasty |
| *Peacock et al^20^* | Day case surgical unit | Orthopaedic surgery | Total joint arthroplasty (hip and knee) |
| *Seetharam et al^32^* | Day case surgical unit | Orthopaedic surgery | Shoulder arthroplasty |
| *Yeung et al^34^* | Day case surgical unit | General surgery | Colorectal and proctology procedures |
| *Beninato et al^3^* | Day case surgical unit | Endocrine surgery | Total thyroidectomy |
| *Cherry et al^35^* | Day case surgical unit | Orthopaedic surgery | Hip and knee arthroplasty |
| *Vogel et al^37^* | Day case surgical unit | NA | Port-a-Cath procedure (insertion or removal) |
| *Thompson et al^51^* | Day case surgical unit | Orthopaedic surgery | Total knee arthroplasty |
| *Brown et al^15^* | Day case surgical unit | Plastic surgery | Aesthetic plastic surgery |
| *Boettner et al ^5^* | Day case surgical unit | Orthopaedic surgery | Arthroplasty surgery |
| *Saravana-Bawan et al^9^* | Day case surgical unit | General surgery | Parathyroidectomy |
| *Grubbs et al^19^* | Day case surgical unit | General surgery | Bariatric surgery |
| *Magruder et al^49^* | Day case surgical unit | Orthopaedic surgery | Uni-compartmental knee arthroplasty |
| *Abdelaal et al^50^* | Day case surgical unit | Orthopaedic surgery | Total hip and knee arthroplasty |
| *Ong et al^2^* | Extension of surgical activity | General surgery, Orthopaedic surgery, Gynaecology, Urology, Plastic surgery, Dental surgery | Laparoscopic cholecystectomy, umbilical / inguinal hernia repairs, excision of pilonidal sinus, laparotomy and small bowel resection, endoscopy, colonoscopy, haemorrhoid artery ligation/ haemorrhoidectomy, laparoscopic right hemicolectomy, examination under anaesthesia, closure of ileostomy, carpal tunnel release, orchidectomy, cystoscopy / pyeloscopy/ ureteroscopy, transurethral resection of bladder tumour/ prostate, ureteric stent placement, excision of squamous cell carcinoma of ear with FLAP, excision of recurrent infra-auricular basal cell carcinoma, superficial lower parotidectomy and cervicofacial FLAP, division of left lower lid chondro-mucosal FLAP with insetting, open reduction internal fixation, anterior cruciate ligament reconstruction arthroscopies, rotator cuff repair, meniscal repair/ debridement, cystectomy, hysteroscopy + dilatation and curettage excision of endometriosis, *Mirena* insertion, hysterectomy, salpingectomy, oophorectomy, myomectomy, anterior / posterior vaginal repair, left revision mastoidectomy, tonsillectomy |
| *Matava et al^21^* | Extension of surgical activity, Day case surgical unit | Plastic surgery, Urology, Dental surgery, Ophthalmology, Otolaryngology | Amputation sixth digit/polydactyly excision, Coleman fat transfer/fat injection, cyst/lesion/skin tag excision, duplicated digit reconstruction, excisional biopsy, haemangioma/mixed capillary and lymphatic malformation excision, nevus excision, plate and screw removal, scar tissue revision, setback otoplasty (5 bilateral, 1 unilateral), subungual exostosis excision, tongue tie release, trigger finger/thumb release, hydrocele repair, orchidopexy, orchiectomy, penoplasty, dental extraction and restoration, botulinum injection, cataract extraction, conjunctival biopsy, corneal crosslinking/revision, entropion repair, ocular imaging / fluorescein angiogram, eyelid dermoid excision, myectomy, nystagmus surgery, orbital dermoid cyst excision, ptosis repair, strabismus repair, rectus recession, tear duct probe, cochlear implant, fess, polypectomy, maxillary enterostomy, ethmoidectomy, sphenoidotomies, frontal, sinusotomy, tympanoplasty |
| *Veraldi et al^30^* | Integrated hub | Vascular surgery | Endovascular procedures, open procedures, hybrid procedures, major limb amputation |
| *Mamidanna et al^13^* | Integrated hub | General surgery | Bariatric surgery |
| *Huddy et al (b)^39^* | Integrated hub | Colorectal surgery, Urology | Robotic colorectal and urological cancer surgery |
| *Pelle et al^46^* | Integrated hub | Breast surgery | Breast cancer surgery |
| *Huddy et al (a)^6^* | Integrated hub | General surgery, Urology, Vascular surgery | Robotic colorectal and urological cancer surgery and regional vascular surgery |
| *Jeannon et al^40^* | Integrated hub, public-private hub | ENT surgery | Partial glossectomy neck dissection + free flap, partial glossectomy or floor of mouth resection + primary closure, wide local excision of skin + free flap, total thyroidectomy + neck dissection, thyroid lobectomy, pan endoscopy (diagnostic), total parotidectomy, transoral robotic surgery, lateral temporal bone resection + flap, transoral laser microsurgery |
| *Friebel et al^23^* | Public-private hub | All surgical specialties | NA |
| *Sairally and Clark^52^* | Public-private hub | Gynaecology | NA |
| *Prokopenko and Khatkar^41^* | Public-private hub | Orthopaedic surgery | Ankle ORIF, distal radius ORIF, total hip replacement, dynamic hip screw, hemiarthroplasty |
| *Barker et al^43^* | Public-private hub | Breast, ENT surgery, General surgery, Gynaecology, Trauma and urgent spinal surgery, Plastic surgery, Urology, Vascular surgery | NA |
| *Collins et al^17^* | Public-private hub | Urology | Nephrectomy, cystectomy, TURBT, RARP, orchidectomy, FURS, rigid cystoscopy procedures |
| *Margaret K. Ho^10^* | Public-private hub | Orthopaedic surgery | Total joint replacement surgery |
| *Hampton et al^48^* | Public-private hub, day case surgical unit | Orthopaedic surgery | Hip and knee arthroplasty |
| *Rafaih Iqbal et al^45^* | Public-private hub, day case surgical unit | Breast surgery, General surgery, Urology, ENT surgery, Gynaecology, Maxillo-facial surgery | NA |
| *Lin et al^7^* | Staff capacity expansion | Ophthalmology | Cataract surgery |
| *McCabe et al^44^* | Staff capacity expansion, public private hub | All surgical specialties | NA |
| *Chu et al^25^* | Stand-alone hub | ENT surgery | Head and neck cancer surgery |
| *Phelan et al^28^* | Stand-alone hub | General surgery, Urology and ENT surgery | Major surgery (defined as the need of PACU postoperatively) |
| *Milito et al^29^* | Stand-alone hub | Thoracic surgery, General surgery | Esophagectomy MIS |
| *Carvalho et al^31^* | Stand-alone hub | General surgery | Colorectal cancer surgery and anal cancer surgery |
| *AlShareef et al^33^* | Stand-alone hub | All surgical specialties | NA |
| *Chuntamongkol et al^12^* | Stand-alone hub | Orthopaedic surgery | Knee and hip surgery |
| *Craig et al^36^* | Stand-alone hub | Ophthalmology, Plastic surgery | Hand surgery and cataract surgery |
| *Chong et al^38^* | Stand-alone hub | Cardiac surgery, Thoracic surgery, Breast surgery, Gastrointestinal surgery, Gynaecology, Head and neck surgery, Hepatobiliary surgery, Neurosurgery, Plastic surgery, Urology, Vascular surgery | NA |
| *Spinelli et al^14^* | Stand-alone hub | General surgery | Oncologic colorectal surgery |
| *Lee et al (AlShareef et al)^1^* | Stand-alone hub | Gynaecology, Urology, Orthopaedic surgery, General surgery, Cardiothoracic surgery, Ophthalmology, Colorectal surgery, Breast surgery, Vascular surgery, Plastic surgery, ENT surgery, Hepatobiliary/Pancreatic surgery | NA |
| *Dafydd et al^42^* | Stand-alone hub | Breast surgery, Gynaecology, Head and neck surgery, Lower gastrointestinal surgery, Thoracic surgery, Hepato-pancreato-biliary surgery, Urology, Sarcoma surgery, Endocrine surgery, Plastic surgery | Oncologic surgery |
| *Chessa et al^4^* | Stand-alone hub | Cardiac surgery | Congenital heart disease cardiac surgery (5 neonatal cardiac surgeries and 16 paediatric cardiac surgeries) |
| *Hanrahan et al^53^* | Stand-alone hub | Neurosurgery | NA |
| *Sorrentino et al^8^* | Stand-alone hub | General surgery | Colorectal cancer surgery |
| *Antonio Maria et al^47^* | Stand-alone hub | Urology | Laparoscopic radical prostatectomy, laparoscopic radical cystectomy, laparoscopic nephrectomy, transurethral resection of bladder tumour, ureter lithotripsy |
| *Doyle et al^18^* | Stand-alone hub | General surgery | Ivor-Lewis oesophagectomy, oesophagogastrectomy with right lower lobectomy, total gastrectomy, subtotal gastrectomy, GIST resection, Whipple procedure, distal pancreatic resection, total pancreatectomy, other pancreatic resections, major and minor liver resections, cholecystectomy, small bowel resection, adrenalectomy, splenectomy, excision of complex mediastinal mass, excision of diaphragmatic tumour, de-roofing of giant liver cyst |
| *Kasivisvanathan et al^11^* | Stand-alone hub, public-private hub | NA | Cancer surgery |
| *Perrone et al^16^* | Stand-alone hub, public-private hub | Gynaecology | Tumour resection (uterine, vulvar, ovarian, breast) |

Table S6: Strengthening of system preparedness indicators by this framework

Social support, remote outpatients, family communication, preoperative assessment, electricity supply, oxygen supply, drug supply, device supply, sterilisation, protective equipment, patient prioritisation, and procedure prioritisation were considered outside of the boundaries of this framework, therefore, these were not included in the table above.

References

1. Lee G, Clough OT, Walker JA, Anakwe RE. The perception of patient safety in an alternate site of care for elective surgery during the first wave of the novel coronavirus pandemic in the United Kingdom: a survey of 158 patients. *Patient Saf Surg*. Mar 12 2021;15(1):11. doi:10.1186/s13037-021-00284-8

2. Ong BS, Thomas R, Jenkins S. Introducing the "Twilight" operating room concept: a feasibility study to improve operating room utilization. *Patient Saf Surg*. Jul 27 2022;16(1):23. doi:10.1186/s13037-022-00335-8

3. Beninato T, Laird AM, Graves CE, et al. Impact of the COVID-19 pandemic on the practice of endocrine surgery. *Am J Surg*. Apr 2022;223(4):670-675. doi:10.1016/j.amjsurg.2021.07.009

4. Chessa M, Varrica A, Andronache A, et al. Lombardy regional urgent reorganization for congenital cardiac patients following the Covid-19 pandemic. *J Cardiovasc Med (Hagerstown)*. Sep 2020;21(9):654-659. doi:10.2459/JCM.0000000000001055

5. Boettner F, Bostrom MP, Figgie M, et al. Timeline and Procedures on Restarting Non-Emergent Arthroplasty Care in the US Epicenter of the COVID-19 Pandemic. *HSS J*. Nov 2020;16(Suppl 1):146-152. doi:10.1007/s11420-020-09801-4

6. Huddy JR, Freeman Z, Crockett M, et al. Establishing a "cold" elective unit for robotic colorectal and urological cancer surgery and regional vascular surgery following the initial COVID-19 surge. *Br J Surg*. Oct 2020;107(11):e466-e467. doi:10.1002/bjs.11922

7. Lin PF, Naveed H, Eleftheriadou M, Purbrick R, Zarei Ghanavati M, Liu C. Cataract service redesign in the post-COVID-19 era. *Br J Ophthalmol*. Jun 2021;105(6):745-750. doi:10.1136/bjophthalmol-2020-316917

8. Sorrentino L, Guaglio M, Cosimelli M. Elective colorectal cancer surgery at the oncologic hub of Lombardy inside a pandemic COVID-19 area. *J Surg Oncol*. Aug 2020;122(2):117-119. doi:10.1002/jso.26052

9. Saravana-Bawan B, Auguste BL, Zahirieh A A Devon K. Ambulatory Parathyroidectomy for Secondary Hyperparathyroidism at a Large Dialysis Program in Toronto: A Program Report. Can J Kidney Health Dis. 2022. 10.1177/20543581221127937

10. Ho MK. Total joint replacement surgeries: Making the case for a public–private partnership in Hong Kong. *World Medical & Health Policy*. 2021;14(3):600-608. doi:10.1002/wmh3.467

11. Kasivisvanathan R, Tilney HS, Jhanji S, et al. The 'hub and spoke model' for the management of surgical patients during the COVID-19 pandemic. *Int J Health Plann Manage*. Sep 2021;36(5):1397-1406. doi:10.1002/hpm.3243

12. Chuntamongkol R, Meen R, Nash S, Ohly NE, Clarke J, Holloway N. Resuming elective orthopaedic services during the COVID-19 pandemic : our experience. *Bone Jt Open*. Nov 2021;2(11):951-957. doi:10.1302/2633-1462.211.BJO-2021-0080.R1

13. Mamidanna R, Askari A, Patel K, et al. Safety and feasibility of resuming bariatric surgery under the cloud of COVID-19. *Ann R Coll Surg Engl*. Jul 2021;103(7):524-529. doi:10.1308/rcsann.2021.0053

14. Spinelli A, Carvello M, Carrano FM, et al. Reduced duration of stay after elective colorectal surgery during the peak phase of COVID-19 pandemic: A positive effect of infection risk awareness? *Surgery*. Aug 2021;170(2):558-562. doi:10.1016/j.surg.2020.12.017

15. Brown M, Eardley S, Ahmad J, et al. The Safe Resumption of Elective Plastic Surgery in Accredited Ambulatory Surgery Facilities During the COVID-19 Pandemic. *Aesthet Surg J*. Oct 15 2021;41(11):NP1427-NP1433. doi:10.1093/asj/sjaa311

16. Perrone AM, Dondi G, Giunchi S, et al. COVID-19 free oncologic surgical hub: The experience of reallocation of a gynecologic oncology unit during pandemic outbreak. *Gynecol Oncol*. Apr 2021;161(1):89-96. doi:10.1016/j.ygyno.2020.09.030

17. Collins PM, Madden A, O'Connell C, et al. Urological service provision during the COVID-19 period: the experience from an Irish tertiary centre. *Ir J Med Sci*. May 2021;190(2):455-460. doi:10.1007/s11845-020-02352-x

18. Doyle JP, Patel PH, Doran SLF, et al. The Cancer Hub Approach for Upper Gastrointestinal Surgery During COVID-19 Pandemic: Outcomes from a UK Cancer Centre. *Ann Surg Oncol*. Apr 2023;30(4):2266-2275. doi:10.1245/s10434-022-12571-4

19. Grubbs JE, Daigle HJ, Shepherd M, et al. Fighting the obesity pandemic during the COVID-19 pandemic. *Surg Endosc*. Sep 26 2022:1-7. doi:10.1007/s00464-022-09628-6

20. Peacock S, Wolfstadt J, Peer M, Gleicher Y. Rapid implementation of an outpatient arthroplasty care pathway: a COVID-19-driven quality improvement initiative. *BMJ Open Qual*. Mar 2022;11(1)doi:10.1136/bmjoq-2021-001698

21. Matava C, So J, Williams RJ, Kelley S, Group OR-X. A Canadian Weekend Elective Pediatric Surgery Program to Reduce the COVID-19-Related Backlog: Operating Room Ramp-Up After COVID-19 Lockdown Ends-Extra Lists (ORRACLE-Xtra) Implementation Study. *JMIR Perioper Med*. Mar 15 2022;5(1):e35584. doi:10.2196/35584

22. Hammerberg EM, Tucker NJ, Stacey SC, et al. Institution of same-day total joint replacement at an urban safety net hospital during the COVID-19 pandemic. *J Orthop*. Nov-Dec 2022;34:173-177. doi:10.1016/j.jor.2022.08.029

23. Friebel R, Fistein J, Maynou L, Anderson M. Emergency contracting and the delivery of elective care services across the English National Health Service and independent sector during COVID-19: a descriptive analysis. *BMJ Open*. Jul 18 2022;12(7):e055875. doi:10.1136/bmjopen-2021-055875

24. Tran-McCaslin M, Basam M, Rudikoff A, Thuraisingham D, McLemore EC. Reduced Opioid Use and Prescribing in a Same Day Discharge Pilot Enhanced Recovery Program for Elective Minimally Invasive Colorectal Surgical Procedures During the COVID-19 Pandemic. *Am Surg*. Oct 2022;88(10):2572-2578. doi:10.1177/00031348221109467

25. Chu F, Zocchi J, De Berardinis R, et al. COVID-19 and head and neck cancer management. Experience of an oncological hub comprehensive cancer centre and literature review. *Acta Otorhinolaryngol Ital*. Apr 2022;42(Suppl. 1):S79-S86. doi:10.14639/0392-100X-suppl.1-42-2022-09

26. Cannon DJ, Lewis S, Garcia J, Watkins A, Rodriguez HC, Levy JC. A comparison of patient same-day discharge selection after shoulder arthroplasty before and after the COVID-19 pandemic. *Semin Arthroplasty*. Sep 2022;32(3):559-563. doi:10.1053/j.sart.2022.02.011

27. Gordon AM, Sheth B, Conway C, Magruder M, Sadeghpour R, Choueka J. The resiliency of elective total shoulder arthroplasty case volumes in the United States during the COVID-19 pandemic: a nationwide temporal trends analysis. *J Shoulder Elbow Surg*. Oct 2022;31(10):e507-e517. doi:10.1016/j.jse.2022.02.045

28. Phelan L, Digne-Malcolm H, Hassett D, Naumann DN, Dilworth MP, Bowley DM. Establishing a COVID-secure site for elective surgery during the COVID pandemic: An observational study. *J Perioper Pract*. Mar 24 2022:17504589211031083. doi:10.1177/17504589211031083

29. Milito P, Asti E, Resta M, Bonavina L. Minimally invasive esophagectomy for cancer in COVID hospitals and oncological hubs: are the outcomes different? *Eur Surg*. 2022;54(2):98-103. doi:10.1007/s10353-022-00751-1

30. Veraldi GF, Mezzetto L, Perilli V, et al. Clinical and Economic Impact of COVID-19 in Vascular Surgery at a Tertiary University "Hub" Hospital of Italy. *Ann Vasc Surg*. Jul 2022;83:97-107. doi:10.1016/j.avsg.2022.02.004

31. Carvalho F, Rogers AC, Chang TP, et al. Feasibility and usability of a regional hub model for colorectal cancer services during the COVID-19 pandemic. *Updates Surg*. Apr 2022;74(2):619-628. doi:10.1007/s13304-022-01264-y

32. Seetharam A, Ghosh P, Prado R, Badman BL. Trends in outpatient shoulder arthroplasty during the COVID-19 (coronavirus disease 2019) era: increased proportion of outpatient cases with decrease in 90-day readmissions. *J Shoulder Elbow Surg*. Jul 2022;31(7):1409-1415. doi:10.1016/j.jse.2021.12.031

33. AlShareef Y, AlShammary SA, Abuzied Y, AlAsseri Y, AlQumaizi KI. Assigning green hospitals during the COVID-19 pandemic assure continuous and safe resumption of surgical services. *Ann Med Surg (Lond)*. Jan 2022;73:103207. doi:10.1016/j.amsu.2021.103207

34. Yeung T, Merchant J, Chen P, et al. The impact and restoration of colorectal services during the coronavirus disease 2019 pandemic: A view from Oxford. *Surg Pract*. Feb 2022;26(1):27-33. doi:10.1111/1744-1633.12531

35. Cherry A, Montgomery S, Brillantes J, et al. Converting hip and knee arthroplasty cases to same-day surgery due to COVID-19. *Bone Jt Open*. Jul 2021;2(7):545-551. doi:10.1302/2633-1462.27.BJO-2021-0029.R1

36. Craig JE, Martin-Krajewski CA, Bledsoe JM, et al. Regional Specialty Surgical Practice Efficiencies Gained as a Result of COVID-19. *Mayo Clin Proc Innov Qual Outcomes*. Aug 2021;5(4):693-699. doi:10.1016/j.mayocpiqo.2021.06.003

37. Vogel T, Schippers D, Aldarweesh B, et al. Effective operating room (OR) utilization by performing low-complex surgical procedures during the 2020 corona pandemic. *Int J Comput Assist Radiol Surg*. Aug 2021;16(8):1357-1359. doi:10.1007/s11548-021-02392-3

38. Chong S, Hung R, Gwozdz A, et al. 30-Day postoperative COVID-19 outcomes in 398 patients from regional hospitals utilising a designated COVID-19 minimal surgical site pathway. *Ann R Coll Surg Engl*. Jun 2021;103(6):395-403. doi:10.1308/rcsann.2020.7072

39. Huddy JR, Crockett M, Nizar AS, et al. Experiences of a "COVID protected" robotic surgical centre for colorectal and urological cancer in the COVID-19 pandemic. *J Robot Surg*. Feb 2022;16(1):59-64. doi:10.1007/s11701-021-01199-3

40. Jeannon JP, Simo R, Oakley R, et al. Head and neck cancer surgery during the coronavirus pandemic: a single-institution experience. *J Laryngol Otol*. Feb 2021;135(2):168-172. doi:10.1017/S0022215121000426

41. Prokopenko M, Khatkar H. A District General Hospital Trauma Service Response to COVID-19: Lessons Learnt. *Cureus*. Dec 14 2020;12(12):e12087. doi:10.7759/cureus.12087

42. Ap Dafydd D, O'Mahony M, Jhanji S, et al. The role of CT chest in screening for asymptomatic COVID-19 infection in self-isolating patients prior to elective oncological surgery: findings from a UK Cancer Hub. *Br J Radiol*. Jan 1 2021;94(1117):20200994. doi:10.1259/bjr.20200994

43. Barker T, Barker P, Sokalsky L, McNamara I. The use of the independent sector in providing NHS services during the Covid-19 outbreak; two hospitals experience. *Surgeon*. Oct 2021;19(5):e213-e216. doi:10.1016/j.surge.2020.09.009

44. McCabe R, Schmit N, Christen P, et al. Adapting hospital capacity to meet changing demands during the COVID-19 pandemic. *BMC Med*. Oct 16 2020;18(1):329. doi:10.1186/s12916-020-01781-w

45. Iqbal MR, Dhahri AA, Darwish NMM, Vijay V. Single centre concept of 'cold site' elective surgery during the peak of COVID-19 pandemic : A cohort study. *Ann Med Surg (Lond)*. Nov 2020;59:245-250. doi:10.1016/j.amsu.2020.09.047

46. Pelle F, Cappelli S, Graziano F, et al. Breast cancer surgery during the Covid-19 pandemic: a monocentre experience from the Regina Elena National Cancer Institute of Rome. *J Exp Clin Cancer Res*. Aug 27 2020;39(1):171. doi:10.1186/s13046-020-01683-y

47. Antonio Maria G, Vasileios P, Giacomo Piero I, et al. Urologic surgery and invasive procedures during coronavirus pandemic: Retrospective comparison of risk infection in a referral Covid hospital and in a free-Covid hospital. *Urologia*. May 10 2020;87(4):391560320927106. doi:10.1177/0391560320927106

48. Hampton M, Riley E, Garneti N, Anderson A, Wembridge K. The orthopaedic waiting list crisis : two sides of the story. *Bone Jt Open*. Jul 2021;2(7):530-534. doi:10.1302/2633-1462.27.BJO-2021-0044.R1

49. Magruder ML, Gordon AM, Sheth BK, Conway CA, Wong CHJ. The effects of the COVID-19 pandemic on elective unicompartmental knee arthroplasty in the USA: further evidence that outpatient arthroplasty is safe and effective. *Eur J Orthop Surg Traumatol*. Sep 17 2022:1-8. doi:10.1007/s00590-022-03393-x

50. Abdelaal MS, Small I, Sherman MB, Courtney PM, Sharkey PF. One Year Later: The Lasting Effect of the COVID-19 Pandemic on Elective Hip and Knee Arthroplasty. *J Am Acad Orthop Surg*. Nov 15 2022;30(22):e1474-e1482. doi:10.5435/JAAOS-D-22-00245

51. Thompson JW, Wignadasan W, Ibrahim M, Plastow R, Beasley L, Haddad FS. The introduction of day-case total knee arthroplasty in a national healthcare system: A review of the literature and development of a hospital pathway. *Surgeon*. Apr 2022;20(2):103-114. doi:10.1016/j.surge.2021.01.017

52. Sairally BZF, Clark TJ. Prioritisation of outpatient appointments and elective surgery in gynaecology. *Best Pract Res Clin Obstet Gynaecol*. Jun 2021;73:2-11. doi:10.1016/j.bpobgyn.2021.03.002

53. Hanrahan JG, Burford C, Adegboyega G, et al. Early Responses of Neurosurgical Practice to the Coronavirus Disease 2019 (COVID-19) Pandemic: A Rapid Review. *World Neurosurg*. Sep 2020;141:e1017-e1026. doi:10.1016/j.wneu.2020.06.167
